# Religious Belief and Social Support Among Cancer Patients in Saudi Arabia

**DOI:** 10.7759/cureus.7012

**Published:** 2020-02-16

**Authors:** Abdullah Bany Hamdan, Fawzi Al-odeh, Sherwynn Javison, Abdullah AlSuheil, Sami Alshammary, Musa AlHarbi

**Affiliations:** 1 Oncology, Comprehensive Cancer Center, King Fahad Medical City, Riyadh, SAU; 2 School of Advanced Studies, Saint Louis University, Baguio, PHL; 3 Palliative Care, Comprehensive Cancer Center, King Fahad Medical City, Riyadh, SAU

**Keywords:** cancer, oncology, belief, coping, counselor, spirituality

## Abstract

Background

People with cancer usually experience some degree of depression, anxiety, and fear, as if embracing the fact that cancer has become part of their lives. Additionally, religious beliefs can influence a patient's support system, as well as the patient's own emotional response, behavior, and decision-making, which can create a conflict with medical treatment. The objective of this study was to assess cancer patients' religious beliefs and social support.

Methods

A cross-sectional study was conducted in 294 adult patients at the Comprehensive Cancer Center of King Fahad Medical City in Riyadh, Saudi Arabia. Patients were interviewed using the System of Belief Inventory (SBI-15R) questionnaire, and responses were noted in the survey form.

Results

The majority (82.3%) of patients were newly diagnosed with cancer and in the treatment phase, whereas 9.9% were in the metastatic phase. The total mean score of the SBI-15R scale was 27.9. The mean score of the social support subscale was 13.1 ± 1.68, whereas the mean score for the beliefs and practice subscale was 29.7 ± 0.81. For the social support subscale, a statistically significant difference was found in age (P < 0.001), gender (P < 0.001), and occupation (P = 0.009). However, for the beliefs and practice subscale, a statistically significant difference was found only with gender (P = 0.001).

Conclusions

This study concluded that social support is important for the study participants, and they were attached to their beliefs and cultural practices, as indicated by the high total mean score on the SBI-15R. Understanding this topic is important in order for healthcare organizations to provide holistic patient care.

## Introduction

Cancer is a disease that alters life functions and often challenges optimism, well-being, and decision-making [1]. Cancer patients experience varying degrees of depression, anxiety, and fear. A cancer diagnosis creates a concern related to spiritual values and issues that pertain to religion and God [2]. Religion and spirituality affect the degree of coping with the diagnosis and can provide beneficial themes in enhancing the quality of life despite cancer. Religion is a transcendental belief and practice that passes on from one believer to another based on formally documented doctrine or established cultural practices [1,2]. On the other hand, spirituality is an attachment to religious values or a matter of spirit, meaning it is a state of connecting oneself to God, nature, one another, and the deepest parts of ourselves.

However, why do people seek religious help in times of need? Life-threatening and life-limiting situations could lead people to be attached to their God to increase their hope and transcendence of reality. A person can feel physical and emotional weakness, but perhaps their strength is revived through daily adjustment and seeking help from experts, such as support teams. It is evident that religion influences the support system, the patient’s emotional response, behavior, and even survival during illness. It might affect decision-making skills, which can create a conflict with medical treatment [3]. With these conflicting ideas, many organizations across the globe have sought to improve the religion/spirituality process by establishing an assessment tool and structured spiritual services so that conflicts could be avoided or minimized. In Saudi Arabia, hospital spiritual counselors and religious teams are inclined with healthcare sectors to streamline the treatment process without compromising medical interventions. When discussing medical plans, spiritual counselors should be present in patient/family meetings and multidisciplinary meetings, as per the mandate of the Joint Commission on Accreditation of Healthcare Organizations, which states: “For many patients, pastoral care and other spiritual services are an integral part of health care and daily life. The hospital can provide for pastoral care and other spiritual services for patients who request them” [3,4].

Religion and medicine could have a great impact on cancer survival. Researchers who have studied the importance of religion and spirituality in helping patients cope with cancer recently introduced subdimensional areas that could make a clinically ill person cope with their life-seeking burdens. These subdimensions include beliefs, devotional practices, experiences, and relationships, which are the four psych emotional areas linked to the religious and spiritual realm [5].

The National Consensus Project for Quality Palliative Care established national palliative care guidelines that pay attention to patients’ religious and spiritual needs [6]. Most critically ill, dying patients are under palliative care treatment requiring thorough management. Palliative care patients certainly need medical and spiritual management to improve the quality of their day-to-day living [6,7]. Addressing spiritual or religious concerns enables the patient to improve their quality of life and establish hope despite the illness. Additionally, when patients have spiritual and situational support, they are better able to find their peace with God and express their spiritual concerns [1]. However, little is known about religious beliefs and social support among cancer patients in Saudi Arabia. Therefore, this study aimed to assess cancer patients’ religious beliefs and social support at a tertiary care hospital.

## Materials and methods

Study design and population

A cross-sectional study was conducted in the Comprehensive Cancer Center (CCC) of King Fahad Medical City (KFMC) in Riyadh, Saudi Arabia, throughout one year. The respondents were all adult cancer patients (inpatient and outpatient) of CCC who received any means of spiritual counseling. Patients/families who refused, cancer patients outside CCC, comatose and disoriented patients, and patients younger than 14 years were excluded from this study. Adult cancer patients who received spiritual counseling were randomly approached and asked to participate in this study by an invitation letter attached to the questionnaire and cover page explaining the study aim. Written informed consent was signed by all participants who agreed to take part in this study. The study was approved by the Institutional Review Board of KFMC (IRB Number 17-163).

Data collection tool

Data were collected using the System of Belief Inventory (SBI-15R) questionnaire, developed by Holland [8]. It includes 15 close-ended statements, including 10 statements in the beliefs and practice sections and the remaining statements in the social support section. The tool used a four-point Likert scale to determine the actual scores based on an established grading system ranging from strongly disagree (0) to strongly agree (3), and none of the time (0) to all of the time (3). The total scores ranged between 0 and 30 for the beliefs and practice subgroups and between 0 and 15 for the support subgroup, and higher scores indicated a higher level of spiritual engagement. The score for the survey tool could be obtained by adding the two-subgroup scores. Moreover, the demographic and clinical characteristics of the study participants were recorded.

Reliability and validity of the questionnaire

The questionnaire was translated into Arabic, according to the Guidelines for Cross-Cultural Adaptation of Health Questionnaires and Diagnostic Tests [9,10]. The questionnaire was reviewed by multiple expert panels from oncology, spirituality, and biostatistics. A pilot study was conducted for the prefinal version of the questionnaire on 25 participants to measure the validity through the test and retest analysis. The assessments of the reliability and internal consistency of the questionnaire were performed using Cronbach's alpha test, and it was estimated at 0.80.

Sample size and statistical analysis

Cochran’s method was used to determine the sample size with the help of Rao Online Software (Raosoft, Inc. Seattle, WA). We took into account the fact that 50% of patients put their trust in religious and spiritual care. Therefore, based on a 95% confidence interval, there was a 5.3% margin of error and 80% power to detect such difference. An estimated sample of 350 patients was recruited in this study through a convenience sampling procedure. Demographic characteristics and responses of study participants were reported as mean (standard deviation), median (range), or counts (percentage). Differences in SB-15R scores were compared between groups using a t-test, Mann-Whitney U test, and Kruskal-Wallis test, as appropriate. All statistical analyses were performed using IBM SPSS Statistics for Windows, Version 25.0 (Armonk, NY: IBM Corp.), and a two-tailed P-value of 0.05 was considered significant.

## Results

Of the 350 patients who participated in this study, 56 patients were not able to complete the questionnaire. Hence, the statistical analysis was conducted on 294 patients. The median age of study participants was 52 years, and 56.5% were male. The majority (82.3%) of the patients were newly diagnosed with cancer and in the treatment phase, whereas 9.9% were in the metastatic phase (Table 1). 

**Table 1:** Demographic and clinical characteristics of the study participants Data presented either as number and percentage or median and (minimum-maximum).

Variable		
Median age in years (range)		52 (14-88)
Gender	Male	166 (56.5%)
	Female	128 (43.5%)
Department	Psychology	2 (0.7%)
	Spiritual	291 (99.0%)
	Supportive care	1 (0.3%)
Marital status	Married	227 (77.2%)
	Separated/divorced	8 (2.7%)
	Single	46 (15.6%)
	Widowed	13 (4.4%)
Educational level	University degree	96 (32.7%)
	High school	82 (27.9%)
	Middle school	48 (16.3%)
	Others	68 (23.1%)
Occupation	Employed	89 (30.3%)
	Retired	62 (21.1%)
	Self-employed	18 (6.1%)
	Housewife	94 (32.0%)
	Other	31 (10.5%)
Religion	Muslim	293 (99.7%)
	Non-Muslim	1 (0.3%)
Cancer site	Breast	49 (16.7%)
	Colon rectum	39 (13.3%)
	Liver	9 (3.1%)
	Sarcoma	3 (1.0%)
	Lymphoma	73 (24.8%)
	Head and neck	34 (11.6%)
	Other	87 (29.6%)
Phase of disease	Diagnosis treatment	242 (82.3%)
	Metastasis	29 (9.9%)
	Palliative	23 (7.8%)
Received at least one treatment in the last three months (surgery, hormone, chemo or radiotherapy)	Yes	277 (94.2%)
	No	17 (5.8%)
Time since diagnosis (days)		210 (3-7200)

The total mean scores of the SBI-15R subscales and statements are presented in Table 2. The total mean score of the SBI-15R scale was 27.9. The mean score of the social support subscale was 13.1 ± 1.68, whereas the mean score for the beliefs and practice subscale was 29.7 ± 0.81.

**Table 2:** SBI-15R mean scores SBI, System of Belief Inventory.

Statements	Mean	SD
SBI-15R social support subscale	13.1	1.68
I enjoy attending religious lectures.	2.6	0.49
When I need clarification on how to deal with problems related to my disease, I contact the religious scholars.	2.3	0.58
When I feel lonely, I rely on my Lord and seek guidance from the spiritual counselor.	2.9	0.35
I seek out for religious support when needed.	2.6	0.56
I enjoy meeting or often talking with people who share my religious or spiritual beliefs.	2.6	0.55
SBI-15R beliefs and practice subscale	29.7	0.81
Religion is important in my day-to-day life.	3.0	0.08
I feel certain that my disease is from Allah (God).	3.0	0.23
I believe Allah (God) will reward me for my patients on my burden.	3.00	0.06
During times of illness, my religious or spiritual beliefs have been strengthened.	2.9	0.25
I have experienced a sense of hope as a result of my religious or spiritual beliefs.	3.0	0.15
I have experienced peace of mind through my prayers and supplications.	3.0	0.13
One’s life and death are in Allah’s (God) hands.	3.00	0.00
I believe Allah (God) protects me from harm.	3.00	0.06
I pray for help during bad times.	2.91	0.30
Prayer helped me cope during times of serious illness.	2.9	0.32

Table 3 shows the association between the SSI-15R beliefs and practice subscale and social support subscale scores and the characteristics of the study participants. For the social support subscale, a statistically significant difference was found in age (P < 0.001), gender (P < 0.001), and occupation (P = 0.009). On the other hand, for the beliefs and practice subscale, a statistically significant difference was found only with gender (P = 0.001).

**Table 3:** SBI-15R scores by the study sample characteristics SBI, System of Belief Inventory.

		SBI-15R Social Support			SBI-15R Beliefs and Practice		
		Mean (SD)	Median (min-max)	P-value	Mean (SD)	Median (min-max)	P-value
Age (years)	<52	12.7 (1.85)	13.0 (8, 15)	0.001	29.5 (0.99)	30.0 (25, 30)	0.001
	≥52	13.4 (1.42)	14.0 (9, 15)		29.8 (0.55)	30.0 (26, 30)	
Gender	Male	13.4 (1.47)	14.0 (8, 15)	<0.001	29.7 (0.81)	30.0 (25, 30)	0.29
	Female	12.63 (1.84)	13.0 (8, 15)		29.6 (0.82)	30.0 (26, 30)	
Phase of disease	Diagnosis treatment	13.1 (1.72)	13.0 (8, 15)	0.87	29.6 (0.82)	30.0 (25, 30)	0.98
	Metastasis	13.1 (1.54)	14.0 (10, 15)		29.6 (0.94)	30.0 (26, 30)	
	Palliative	13.0 (1.48)	13.0 (10, 15)		29.7 (0.54)	30.0 (28, 30)	
Treatment	Yes	13.1 (1.71)	13.0 (8, 15)	0.23	29.7 (0.82)	30.0 (25, 30)	0.94
	No	12.8 (1.19)	12.0 (11, 15)		29.7 (0.59)	30.0 (28, 30)	
Civil status	Married	13.1 (1.67)	13.0 (8, 15)	0.71	29.7 (0.83)	30.0 (25, 30)	0.87
	Separated/divorced	12.5 (2.56)	13.5 (8, 15)		29.9 (0.35)	30.0 (29, 30)	
	Single	12.9 (1.73)	13.0 (8, 15)		29.6 (0.86)	30.0 (26, 30)	
	Widowed	13.6 (0.96)	14.0 (12, 15)		29.8 (0.44)	30.0 (29, 30)	
Education	University degree	13.1 (1.76)	13.0 (8, 15)	0.21	29.6 (0.87)	30.0 (25, 30)	0.36
	High school	12.7 (1.77)	13.0 (8, 15)		29.6 (0.93)	30.0 (25, 30)	
	Middle school	13.0 (1.87)	13.0 (8, 15)		29.7 (0.86)	30.0 (26, 30)	
	Others	13.4 (1.23)	13.5 (10, 15)		29.8 (0.47)	30.0 (28, 30)	
Occupation	Employed	13.2 (1.71)	14.0 (8, 15)	0.004	29.5 (1.08)	30.0 (25, 30)	0.46
	Retired	13.5 (1.52)	14.0 (9, 15)		29.8 (0.46)	30.0 (28, 30)	
	Self-employed	13.4 (1.46)	13.5 (11, 15)		29.7 (0.67)	30.0 (28, 30)	
	Housewife	12.7 (1.71)	13.0 (8, 15)		29.7 (0.76)	30.0 (26, 30)	
	Other	12.6 (1.67)	13.0 (8, 15)		29.7 (0.66)	30.0 (28, 30)	

## Discussion

This study provided several valuable findings regarding perceptions towards religious beliefs and practice, and social support for patients in a cancer center in Saudi Arabia. This study indicated that patients were more attached to their beliefs and cultural practices in times of life-changing circumstances, as indicated by the high total mean score of the SBI-15R questionnaire and subscales. Additionally, the majority of patients expressed that they felt certain about their disease is from Allah (3.0 ± 0.23) and that they felt a sense of hope as a result of their religious or spiritual beliefs (3.0 ± 0.15). Furthermore, patients sought guidance from their spiritual counselor when they felt lonely (2.9 ± 0.35).

Similarly, previous studies have indicated that religious beliefs and practice and social support are intricate processes, and the ability to assess these areas is vital because 50% to 95% of cancer patients perceived it as important [1,11,12]. A study in Italy was conducted to measure religious and spiritual beliefs and practice as well as social support, and it revealed that religiousness was significantly linked in belief and social support subscales [3]. A study was carried out in the United States to examine religiousness and spiritual support in advanced cancer patients, and it reported that 96% of their adult population expressed a belief in God, while 70% conveyed that religion influenced their lives. Moreover, the study revealed that 49% of women with gynecologic cancer became more religious after the disclosure of their diagnosis [1]. Another study conducted to explore spiritual care needs reported that spiritual care is neglected in many countries because many healthcare professionals are not aware of this need [13]. Furthermore, the study suggested that when health professionals include assessment of religious and spiritual beliefs during a patient interview, it might influence treatment decisions [14]. In another study that was conducted to determine the importance of faith regarding treatment decisions in 100 patients who were newly diagnosed with lung cancer, patients rated their belief in God as the second most important factor in their treatment decision [15].

Moreover, our results showed that on the SBI-15R social support subscale, there is an association between the age, gender, and occupation of the study participants, whereas, for the SBI-15R beliefs and practice subscale, a statistically significant difference was found only with gender.

Our findings provide evidence that spiritual care, religious beliefs and practice, and social support are essential for a cancer patient. Additionally, this study will help organizations to understand the importance of religious beliefs and practice and social support among cancer patients. Establishing a religious program is a vital part of a multidisciplinary approach to improving the care process, as well as the patient and family experience. Programs can include but are not limited to educational workshops, training/courses, and hiring religious counselors and others. The limitations of this study were that it was conducted at a single tertiary care hospital and the findings cannot be generalized to the whole cancer population.

Clinical implications

Spiritual support programs are an essential source of social and faith enhancement and can improve the healthcare process through person-centered care and structured educational programs. Our findings suggest that the application of these types of programs is not only limited to tertiary healthcare institutions, but may also be applicable in secondary and long-term healthcare facilities as well as in non-cancer center institutions. Future research is required to make this study topic robust in Middle East countries [16].

## Conclusions

According to our results, spiritual care is an essential component of overall patient care. Effective strategies and programs to strengthen patient-centered cancer care that focuses on the value of spiritual care and social support are needed. Spiritual support through the enhancement of social well-being might help cancer patients overcome perturbation caused by cancer. Understanding this concept is important for every hospital and medical institution in order to offer more robust, holistic patient care.
